# The association between body roundness index and handgrip strength and muscle quality index: A cross-sectional study

**DOI:** 10.1371/journal.pone.0322928

**Published:** 2025-05-20

**Authors:** Zhihao Wei, Tengfei Yu, Xufeng Jin, Guanyi Ma, Xianfeng Meng

**Affiliations:** 1 Department of Trauma Orthopedics, Shengli Oilfield Central Hospital, Dongying City, Shandong Province, China; 2 Department of Hand and Upper Limb Surgery, Jinan Third People’s Hospital, Jinan City, Shandong Province, China; Tehran University of Medical Sciences, ISLAMIC REPUBLIC OF IRAN

## Abstract

**Background:**

Sarcopenic obesity is characterized by a combination of obesity and sarcopenia. Body round index (BRI) is a novel anthropometric index that can more accurately assess body and visceral fat levels than body mass index or waist circumference. This study used data from the National Health and Nutrition Examination Survey (NHANES) to explore the relationship between BRI and handgrip strength (HGS) and muscle quality index (MQI) in American adults aged 20 and over.

**Methods:**

This study used cross-sectional data from the 2011–2014 National Health and Nutrition Examination Survey (NHANES) with complete data on BRI, HGS, and MQI. We used multivariate linear regression models and smooth curve fitting methods to explore the relationship between BRI and HGS and MQI. In addition, subgroup analyses and interaction tests were performed to further analyze the potential association between these variables.

**Results:**

A total of 5466 participants were finally included in this study, of whom 2807 were males and 2659 were females. The results showed that BRI was positively correlated with HGS and negatively correlated with MQI. In the fully adjusted model, the negative correlation between BRI and MQI was (β= −0.08, 95% CI = −0.08, −0.07), while the positive correlation with HGS was (β= 0.3 8, 95% CI = 0.29, 0.46), indicating that for every unit increase in BRI, MQI decreases by 0.08 units and HGS increases by 0.38 units (P < 0.0001). In addition, the relationship between BRI and HGS is an L-shaped curve. An inflection point is determined when BRI reaches 3.42. Before this threshold, for every unit increase in BRI, HGS increases significantly (β = 2.19, 95% CI = 1.66, 2.72).

**Conclusion:**

The results showed that BRI was positively correlated with HGS and negatively correlated with MQI, meaning that higher BRI was associated with higher HGS and lower MQI. This highlights the importance of body fat distribution in muscle health and suggests that BRI can be used as an effective anthropometric indicator to predict grip strength and muscle mass.

## Introduction

Sarcopenia is a disease of loss of skeletal muscle mass and muscle function commonly seen in older adults [1]. However, recent studies have found that sarcopenia has begun to appear and develop in younger people as well, due to metabolic syndrome, physical inactivity, malnutrition, neuromuscular disorders, and other inflammatory diseases [2]. The Puerto Rican population is the second largest Hispanic subgroup in the United States, with poorer health conditions and higher rates of acute and chronic diseases compared to non-Hispanic whites and other Hispanic subgroups. National data shows that 21% of elderly Puerto Ricans experience restricted mobility. Among young people, the obesity rate of Puerto Rican men was 43% and that of women was 60.5%, higher than that of Mexican Americans of the same age reported by the National Health and Nutrition Examination Survey (NHANES), with the prevalence rate of male obesity of 36.3% and female obesity of 52.2% [3].Exacerbation of sarcopenia not only increases the risk of falls and fractures [4,5], but can also lead to diseases of the medical system and even death [6–8]. This condition is not only a serious health detriment, but also carries a significant personal and socioeconomic burden; among admitted older adults, hospitalization costs for patients with sarcopenia are more than five times higher than for those without sarcopenia [9]. Currently, the treatment of sarcopenia relies on resistance training and aerobic exercise, but no specific drug has been approved for the treatment [10].

Obesity is becoming increasingly prevalent, with the global prevalence of overweight and obesity having doubled since 1980, and about one-third of the world’s population is now classified as overweight or obese [11]. Obesity not only leads to a variety of complications such as diabetes, hypertension, fatty liver and obstructive sleep apnea [12], but is also strongly associated with sarcopenia. Sarcopenic obesity is a condition in which obesity is accompanied by a decrease in lean body mass, defined as the coexistence of obesity with low skeletal muscle mass or function [13]. Traditionally, body mass index (BMI) has been used to define overweight and obesity; however, BMI has low sensitivity and is susceptible to age, gender, and ethnicity, and does not effectively differentiate between muscle and fat mass [14].

The Body Round Index (BRI) is a new geometric index that estimates total and visceral fat by combining height and waist circumference. Compared with traditional BMI, BRI has higher accuracy in predicting body fat and visceral fat percentage [15], providing a new perspective for evaluating body fat percentage and visceral fat ratio. It not only focuses on overall weight, but also more accurately reflects fat distribution patterns [16]. It is now widely used in clinical research and has shown superiority over other human measurement indicators in evaluating various diseases [17–19]. In addition, handgrip strength (HGS) and muscle mass index (MQI) have been widely used to assess the status of sarcopenia [20,21].

To date, no study has determined the relationship between BRI and HGS and MQI. NHANES (National Health and Nutrition Examination Survey) is a nationally representative survey conducted by the Centers for Disease Control and Prevention (CDC) to comprehensively assess the health and nutritional status of the population in the U.S. [22]. The NHANES data, which covers questionnaires, physical examinations, and laboratory tests provides multidimensional health and nutrition indicators and contains information on people of different ages, genders, races, and economic backgrounds, which has high representativeness and application value. Therefore, this study utilized data from the NHANES database with the aim of exploring the association of BRI with HGS and MQI in adults over the age of 20 years.

## Materials and Methods

**Fig 1:**
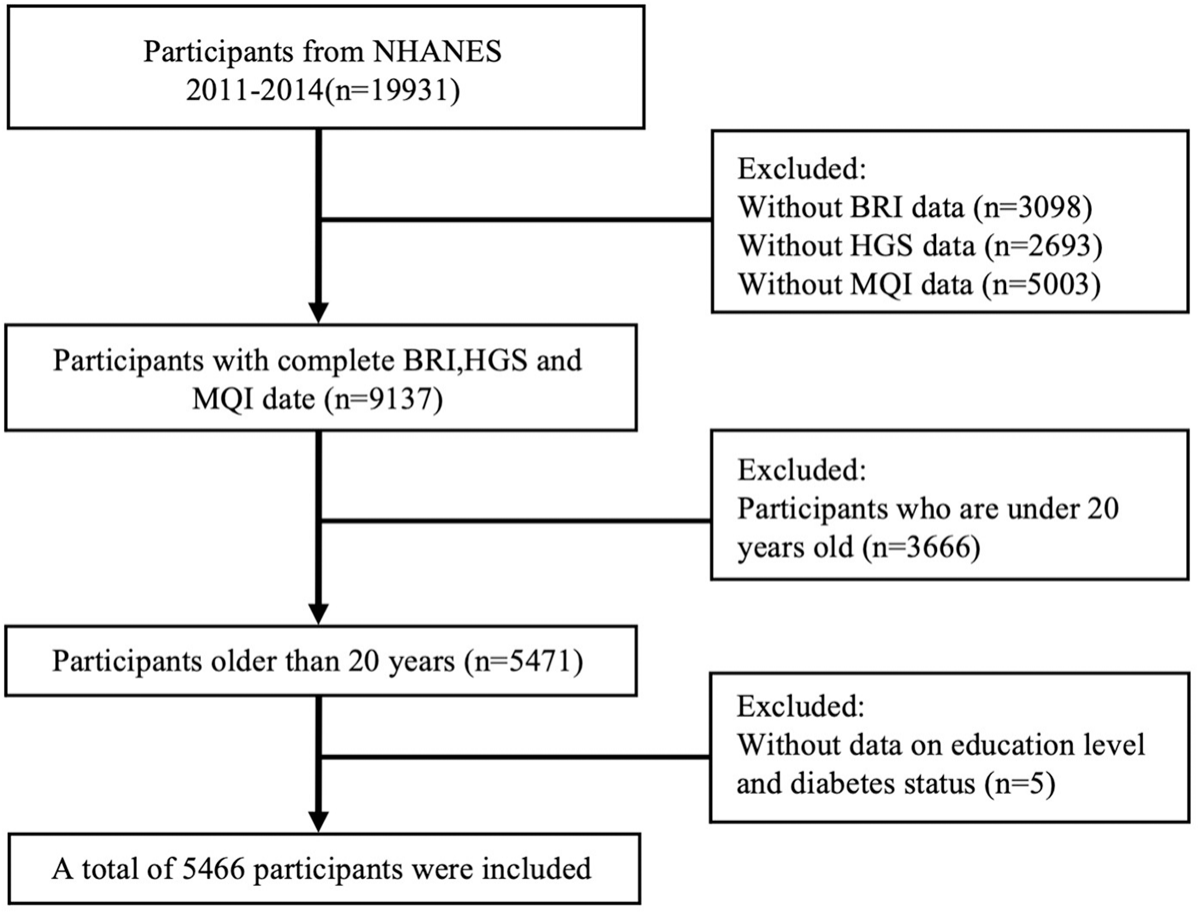
Flow chart of participants selection. NHANES, National Health and Nutrition Examination Survey. BRI body round index, HGS handgrip strength, MQI muscle quality index.

This study utilized data from the NHANES 2011–2014 cycle, with an initial sample size of 19,931 participants. We first excluded 8,602 participants under 20 years of age. Subsequently, we excluded 1,099 participants lacking valid BRI data, 897 participants without HGS measurements, and 3,862 participants missing MQI data. Finally, we excluded participants with missing covariates due to non-response or unknown answers: four participants with undetermined diabetes status and one participant lacking education level information. Ultimately, 5,466 adult participants were included in the analysis. A flowchart illustrating the screening process is presented in Fig 1. The portions of this study involving human participants, human materials, or human data were conducted in accordance with the Declaration of Helsinki and were approved by the NCHS Ethics Review Board. The patients/participants provided their written informed consent to participate in this study.

### Exposed variables and outcome variables

BRI is a novel index for assessing the body fat distribution of an individual, which is calculated from anthropometric data such as waist circumference and height [15]. The formula is: $BRI\;=\;364.2\;-\;365.5\;\times \;\sqrt{}\;[1\;-\;(WC/(2\pi ))2/\;(0.5\;\times \;height)2]$.

HGS was measured using a grip strength meter. All participants with no history of hand or wrist surgery within the past 3 months and no hand pain were eligible to take the test. The test was performed in a standing position and participants were asked to hold the dynamometer as tightly as possible. Each hand was tested three times at 60-second intervals, and the maximum measurement for each hand was used as the final grip strength figure [23].

MQI is defined as the ratio (kg/kg) between the maximum grip strength (HGS, in kilograms) and the attached skeletal muscle mass (ASM) of the limbs [24].

### Covariate

Potential covariates considered in this study included gender, age, race, education level, household income to poverty ratio (PIR), and diabetes status. Continuous variables were age and PIR; categorical variables included gender (female, male), race (non-Hispanic white, non-Hispanic black, Mexican American, etc.), education level (less than 9th grade, 9th to 11th grade, high school graduation or equivalent, partial college or AA degree, college graduation or higher), and diabetes status (absent/present/threshold). Diabetes status was determined by physician diagnosis, use of insulin or diabetes medications, and HbA1c level (≥6.5%).

### Statistical analysis

Continuous variables in this study are expressed as mean ± standard deviation and categorical variables are expressed as frequency (percentage). Between-group comparisons of categorical variables were performed using chi-square tests, and between-group comparisons of continuous variables were performed using analysis of variance (ANOVA) to assess differences in demographic and clinical characteristics between the different BRI groups. Multivariate linear regression models were used to assess the association between BRI and HGS and MQI. Model 1 was an unadjusted model, Model 2 adjusted for age, gender, and race, and Model 3 further adjusted for PIR, education level, and diabetes status based on Model 2. In addition, subgroup analyses were conducted with stratification variables including sex (male/female), age (20–40 years, 40–60 years, > 60 years), race (Mexican American, Other Hispanic, Non-Hispanic White, Non-Hispanic Black, and Other), PIR tertiles, education level (less than 9th grade, 9th grade through 11th grade, high school graduate or equivalent, partial college or AA degree, college graduation or higher) and diabetes (yes/no/ Borderline, as diagnosed by NHANES physicians). Interaction tests were used to assess the consistency of the relationship between age, gender, race, education level, PIR, and diabetes status subgroups. Multivariate analyses were performed by controlling for confounders and fitting smoothed curves. Analyses were performed using R software and Empower software, with statistical significance defined as a p-value below 0.05.

## Results

A total of 5466 participants were finally included in this study, of whom 2807 were males (51.4%) and 2659 were females (48.6%) (Table 1). Participants were divided into three groups based on their BRI levels, with low, medium, and high BRI groups ranging from (1.04–3.83), (3.83–5.56), and (5.56–18.4), respectively. The analysis showed that there were significant differences between the different BRI groups in terms of gender, age, ethnicity, education level, diabetes status, PIR, MQI, and HGS (all p < 0.01). Compared to the low BRI group, the high BRI group had significantly lower mean values for HGS and MQI, and participants were older and had lower PIR (p < 0.01). In addition, the prevalence of diabetes mellitus was 7.3% (n = 400), demonstrating a statistically significant difference in distribution between comparative groups (p < 0.01). Female participants comprised 48.6% (n = 2659) of the population, with similarly significant between-group disparities in gender stratification (p < 0.01).

**Table 1:** Baseline characteristics of participants in the NHANES 2011–2014.

Characteristics	Body round index			P-value
	Low	Medium	High	
Number	1822	1822	1822	
Age(years)	34.60 ± 11.27	40.48 ± 10.96	41.51 ± 11.11	<0.001
PIR	2.62 ± 1.68	2.62 ± 1.65	2.26 ± 1.55	<0.001
MQI (kg/kg)	1.95 ± 0.29	1.80 ± 0.28	1.56 ± 0.28	<0.001
HGS (kg)	40.25 ± 11.42	40.71 ± 11.34	38.69 ± 11.04	<0.001
BRI	2.95 ± 0.58	4.65 ± 0.49	7.60 ± 1.93	<0.001
Gender (%)				<0.001
Male	1032 (56.64%)	1028 (56.42%)	747 (41.00%)	
Female	790 (43.36%)	794 (43.58%)	1075 (59.00%)	
Race (%)				<0.001
Mexican American	108 (5.93%)	263 (14.43%)	319 (17.51%)	
Other Hispanic	138 (7.57%)	165 (9.06%)	184 (10.10%)	
Non-Hispanic White	715 (39.24%)	709 (38.91%)	699 (38.36%)	
Non-Hispanic Black	400 (21.95%)	349 (19.15%)	464 (25.47%)	
Other Race - Including Multi-Racial	461 (25.30%)	336 (18.44%)	156 (8.56%)	
Education level (%)				<0.001
less than 9th grade	48 (2.63%)	107 (5.87%)	112 (6.15%)	
9–11th grade (Includes 12th grade with no diploma)	201 (11.03%)	231 (12.68%)	267 (14.65%)	
high school graduate/GED or equivalent	340 (18.66%)	362 (19.87%)	458 (25.14%)	
some college or AA degree	580 (31.83%)	590 (32.38%)	647 (35.51%)	
college graduate or above	653 (35.84%)	532 (29.20%)	338 (18.55%)	
Diabetes status (%)				<0.001
Yes	35 (1.92%)	110 (6.04%)	255 (14.00%)	
No	1777 (97.53%)	1684 (92.43%)	1517 (83.26%)	
Borderline	10 (0.55%)	28 (1.54%)	50 (2.74%)	

Mean (SD) for continuous variables, % for categorical variables. PIR Ratio of family income to poverty, HGS handgrip strength, MQI muscle quality index, BRI Body round index.

Table 2 shows that in the fully adjusted model, BRI is positively correlated with HGS (β=0.38, 95% CI = 0.29, 0.46) and negatively correlated with MQI (β=-0.08, 95% CI = -0.08, -0.07).In the model without adjustment for covariates (Model 1), higher BRI was significantly associated with lower HGS (β = -0.37, 95% CI = -0.50, -0.24) and MQI (β = -0.08, 95% CI = -0.08, -0.08). The model adjusted for all covariates (Model 3) showed that the negative correlation between BRI and MQI remained significant (β = -0.08, 95% CI = -0.08, -0.07), whereas the relationship with HGS shifted from a negative to a positive correlation (β = 0.38, 95% CI = 0.29, 0.46) indicating that each one-unit increase in BRI was associated with a decrease of 0.08 units in MQI and an increase of 0.38 units in HGS. units and a 0.38 unit increase in HGS was associated. When further comparing between-group differences across BRI tertiles, it was found that the high BRI group had significantly higher HGS and significantly lower MQI than the low BRI group; specifically, the highest BRI tertile group had a 1.94-unit higher HGS than the lowest group (β = 1.94, 95% CI = 1.45, 2.42), whereas the MQI was 0.38 units lower (β = - 0.38, 95% CI = -0.40, -0.36).

**Table 2:** Multivariable logistic regression models for the association between BRI and MQI and HGS.

Exposure	Model 1β (95% CI)	Model 2β (95% CI)	Model 3β (95% CI)
MQI			
BRI	−0.08 (−0.08, −0.08) <0.0001	−0.08 (−0.08, −0.08) <0.0001	−0.08 (−0.08, −0.07) <0.0001
Tertile of BRI			
Tertile 1 (1.04–3.83)	0	0	0
Tertile 2 (3.83–5.56)	−0.15 (−0.17, −0.13) <0.0001	−0.16 (−0.17, −0.14) <0.0001	−0.15 (−0.17, −0.14) <0.0001
Tertile 3 (5.56–18.4)	−0.39 (−0.41, −0.37) <0.0001	−0.39 (−0.41, −0.37) <0.0001	−0.38 (−0.40, −0.36) <0.0001
HGS			
BRI	−0.37 (−0.50, −0.24) <0.0001	0.30 (0.21, 0.38) <0.0001	0.38 (0.29, 0.46) <0.0001
Tertile of BRI			
Tertile 1 (1.04, 3.83)	0	0	0
Tertile 2 (3.83, 5.56)	0.47 (−0.26, 1.20) 0.2107	1.19 (0.72, 1.65) <0.0001	1.24 (0.77, 1.70) <0.0001
Tertile 3 (5.56, 18.4)	−1.56 (−2.29, −0.82) <0.0001	1.62 (1.14, 2.10) <0.0001	1.94 (1.45, 2.42) <0.0001

Model 1: no covariates were adjusted. Model 2: age, gender, and race were adjusted. Model 3: age, gender, race, PIR, education level, and diabetes status. PIR Ratio of family income to poverty, MQI muscle quality index, BRI Body round index, HGS handgrip strength.

In the subgroup analysis (Tables 3 and 4), variables including gender, age, education level, race, and BRI were assessed. Analyses in Tables 3 and 4 revealed significant interactions between BRI and HGS for age (p interaction = 0.0001), education level (p interaction = 0.0111), and race (p interaction = 0.0018). Similarly, BRI and MQI showed significant interactions regarding diabetes status (p interaction = 0.0084), education level (p interaction = 0.0012), and race (p interaction = 0.0338).Specifically, each 1-unit increase in BRI was associated with a 0.5-unit increase in HGS among participants aged <40 years (β = 0.50, 95% CI: 0.38 to 0.62), whereas a 0.17-unit increase was observed in those aged ≥40 years (β = 0.17, 95% CI: 0.05 to 0.30). In patients with diabetes, MQI decreased by 0.06 units per 1-unit increase in BRI (β = -0.06, 95% CI: -0.07 to -0.05), compared to a decrease of 0.08 units in the non-diabetic population (β = -0.08, 95% CI: -0.08 to -0.08). Education-stratified analyses indicated that BRI had the smallest negative effect on MQI and the largest positive effect on HGS in the highly educated group. Race-stratified results demonstrated that a 1-unit BRI increase had the minimal effect on MQI in Non-Hispanic Black individuals and on HGS in Non-Hispanic White individuals. No significant interactions were observed between BRI and HGS (interaction all p = 0.44) or MQI (interaction all p = 0.98) by gender. For other stratified variables, associations of BRI with HGS and MQI were non-significant (all interaction p > 0.05).

**Table 3:** Stratified analysis of the correlation between BRI and MQI.

Subgroup	For MQI		
	**Number**	**[β (95%CI)]**	**P for interaction**
Gender			0.9838
Male	2807	−0.08 (−0.08, −0.07) <0.0001	
Female	2659	−0.08 (−0.08, −0.07) <0.0001	
Age			0.182
Age: < 40	2819	−0.08 (−0.08, −0.07) <0.0001	
Age: >=40	2647	−0.08 (−0.08, −0.07) <0.0001	
Diabetes (%)			0.0084
Diabetes: Yes	400	−0.06 (−0.07, −0.05) <0.0001	
Diabetes: No	4978	−0.08 (−0.08, −0.08) <0.0001	
Diabetes: Borderline	88	−0.07 (−0.09, −0.05) <0.0001	
PIR			0.9558
PIR: low	1811	−0.08 (−0.08, −0.07) <0.0001	
PIR: medium	1829	−0.08 (−0.08, −0.07) <0.0001	
PIR: high	1826	−0.08 (−0.08, −0.07) <0.0001	
Education			0.0012
Less than 9th grade	267	−0.09 (−0.11, −0.07) <0.0001	
9−11th grade (Includes 12th grade with no diploma)	699	−0.09 (−−0.10, −0.08) <0.0001	
High school graduate/GED or equivalent	1160	−0.08 (−0.09, −0.08) <0.0001	
Some college or AA degree	1817	−0.08 (−0.08, −0.07) <0.0001	
College graduate or above	1523	−0.07 (−0.07, −0.06) <0.0001	
Race			0.0338
Mexican American	690	−0.08 (−0.09, −0.07) <0.0001	
Other Hispanic	487	−0.08 (−0.09, −0.07) <0.0001	
Non–Hispanic White	2123	−0.08 (−0.09, −−0.08) <0.0001	
Non–Hispanic Black	1213	−0.07 (−0.08, −0.06) <0.0001	
Other Race - Including Multi-Racial	953	−0.09 (−0.09, −0.08) <0.0001	

Age, gender, race, PIR, education level, and diabetes status were adjusted. PIR Ratio of family income to poverty, HGS handgrip strength, MQI muscle quality index.

**Table 4:** Stratified analysis of the correlation between BRI and HGS.

Subgroup	For HGS		
	Number	[β (95%CI)]	P for interaction
Gender			0.4398
Male	2807	0.42 (0.28, 0.55) <0.0001	
Female	2659	0.35 (0.24, 0.46) <0.0001	
Age			0.0001
Age: < 40	2819	0.50 (0.38, 0.62) <0.0001	
Age: >=40	2647	0.17 (0.05, 0.30) 0.0062	
Diabetes (%)			0.9603
Diabetes: Yes	400	0.38 (0.12, 0.63) 0.0035	
Diabetes: No	4978	0.38 (0.28, 0.47) <0.0001	
Diabetes: Borderline	88	0.29 (−0.31, 0.89) 0.3413	
PIR			0.3423
PIR: low	1811	0.45 (0.32, 0.58) <0.0001	
PIR: medium	1829	0.32 (0.18, 0.47) <0.0001	
PIR: high	1826	0.32 (0.16, 0.49) 0.0001	
Education			0.0111
Less than 9th grade	267	−0.16 (−0.58, 0.27) 0.4614	
9–11th grade (Includes 12th grade with no diploma)	699	0.37 (0.14, 0.59) 0.0014	
High school graduate/GED or equivalent	1160	0.26 (0.09, 0.43) 0.0023	
Some college or AA degree	1817	0.39 (0.26, 0.52) <0.0001	
College graduate or above	1523	0.59 (0.40, 0.78) <0.0001	
Race			0.0018
Mexican American	690	0.27 (0.03, 0.51) 0.0289	
Other Hispanic	487	0.39 (0.10, 0.69) 0.0097	
Non–Hispanic White	2123	0.25 (0.12, 0.38) 0.0002	
Non–Hispanic Black	1213	0.42 (0.26, 0.57) <0.0001	
Other Race - Including Multi-Racial	953	0.82 (0.57, 1.07) <0.0001	

Age, gender, race, PIR, education level, and diabetes status were adjusted. PIR Ratio of family income to poverty, HGS handgrip strength, MQI muscle quality index.

**Fig 2:**
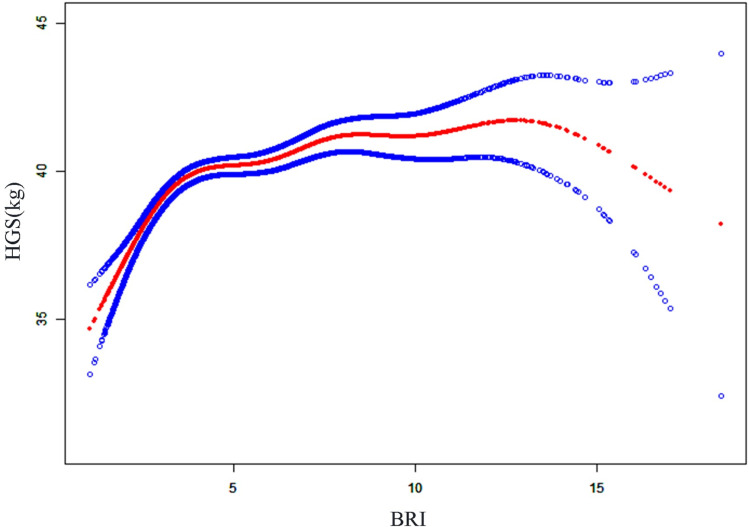
The association between body roundness index and HGS. The solid red line represents the smooth curve fit between variables and the blue bands represent the 95% confidence interval from the fit. HGS handgrip strength, BRI body round index.

**Fig 3:**
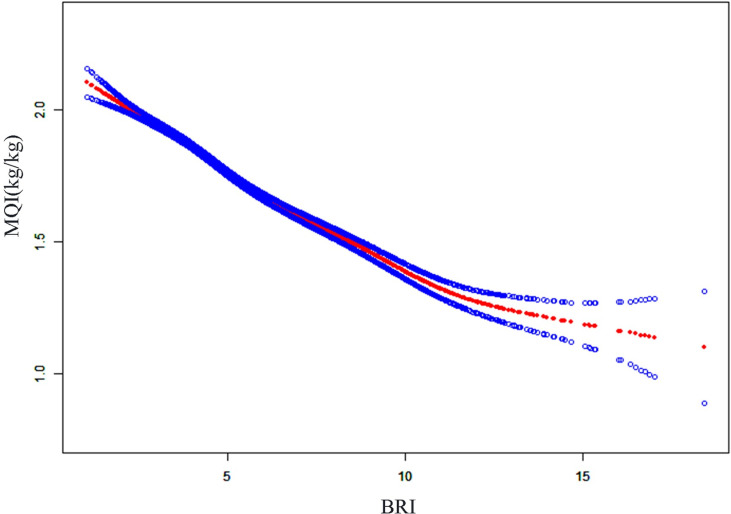
The association between body roundness index and MQI. The solid red line represents the smooth curve fit between variables and the blue bands represent the 95% confidence interval from the fit. MQI muscle quality index, BRI body round index.

In order to explore the nonlinear relationship between BRI and HGS and MQI more comprehensively, this study was analyzed using smooth curve fitting. Figs 2 and 3 showed that BRI was negatively correlated with MQI, whereas it had an L-shaped curve relationship with HGS, with an inflection point of 3.42 (Table 5). Before the inflection point, BRI was significantly positively correlated with HGS (β = 2.19, 95% CI = 1.66, 2.72), and after the inflection point, the two remained positively correlated, but the increase weakened (β = 0.20, 95% CI = 0.10, 0.30).

**Table 5:** The threshold effect analysis of the BRI on HGS.

Outcome	HGS
Model 1 (linear regression)	0.38 (0.29, 0.46) <0.0001
Model 2 (segmented regression)	
Turning point(K)	3.42
BRI < K effect	2.19 (1.66, 2.72) <0.0001
BRI > K effect	0.20 (0.10, 0.30) <0.0001
*P* for log-likelihood ratio	<0.001

Both model 1 and 2 controlled for all covariates. HGS handgrip strength, BRI Body round index.

## Discussion

This study examined the relationship of BRI with HGS and MQI in adults over 20 years of age and found that BRI was positively associated with HGS and negatively associated with MQI. Age, education level, and race showed significant interactions for the relationship between BRI and HGS; whereas the relationship between BRI and MQI showed significant interactions for diabetes status, education level, and race. There was a negative correlation between BRI and MQI, and an L-shaped curvilinear relationship with HGS, with slower growth after the inflection point. The results suggest that increased BRI may be associated with lower MQI and higher HGS.

To our knowledge, there are no comparable studies on the direct relationship between BRI and HGS and MQI. Previous studies have focused on the association of obesity with HGS and MQI. In terms of the relationship between muscle strength and obesity, a cross-sectional study in Taiwan found that higher grip strength was associated with a lower risk of diabetes, suggesting the need to focus on muscle health in obese populations [25]. Another cross-sectional study of 304 participants showed that HGS was positively associated with height and BMI, but was not directly affected by weight, which is consistent with the present study showing that BRI was significantly and positively associated with HGS [26]. This result is supported by a large cross-sectional study based on data from the 1970 British Cohort Study (BCS70), showing that higher BMI since childhood is associated with stronger grip strength at age 46 years, suggesting that the anabolic effects of fat may outweigh its catabolic effects, and thus middle age may be a critical time for intervention to prevent sarcopenic obesity [27]. In terms of the relationship between MQI and obesity, a cross-sectional study including 86 severely obese individuals found that MQI was negatively associated with abdominal obesity and markers of the metabolic syndrome, and mediated the association between abdominal obesity and markers of the metabolic syndrome [28]. Another study found that the negative impact of abdominal obesity on health-related quality of life was partially moderated by MQI [29,30]. In recent years, the impact of obesity on sarcopenia has received increasing attention. A cohort study on sarcopenic obesity and mortality noted that sarcopenic obesity is more prevalent in older adults and is significantly associated with all-cause mortality, a risk that is further exacerbated by changes in body composition. The use of low muscle strength as an initial diagnostic approach may help in the early identification of those at risk [31]. Therefore, considering that BRI, calculated by height and waist circumference, reflects individual body fat distribution, especially abdominal fat, avoids the limitation of BMI that makes it difficult to differentiate between fat and muscle due to the influence of body weight, and is a more effective assessment of visceral fat content than BMI alone, making it a valid measure of obesity. Meanwhile, HGS and MQI are important indicators for assessing sarcopenia, and the results of this study suggest that rational management of BRI may help to improve HGS and MQI to a certain extent, thus contributing to the maintenance and improvement of muscle health.

The present study showed a significant positive relationship between BRI and HGS before the inflection point, indicating that moderate amounts of fat contribute to grip strength. A study on fat-free mice found [32] that muscle mass and strength were significantly reduced in fat-free mice, but muscle function was fully restored after restoring to 10% of normal fat mass, and this restoration was fully mediated by leptin, suggesting that leptin plays a key role in the maintenance of muscle mass and strength in adipose tissue. Another animal experiment showed [33] that in leptin receptor-deficient (Leprdb) mice, the number of skeletal muscle capillaries was significantly reduced, indicating that leptin deficiency affects angiogenesis. Perivascular cells expressing platelet-derived growth factor receptor (PDGFR) β in skeletal muscle may act as “nutrient sensors” to increase leptin production under nutrient-sufficient conditions such as a high-fat diet, thereby elevating the mRNA and protein levels of VEGFA, and linking nutritional status to the angiogenic demands of skeletal muscle. angiogenic requirements. Furthermore, several studies have shown that serum leptin deficiency is detrimental to muscle health [34–36]. Thus, the significant increase in HGS prior to the BRI inflection point may be driven by the supportive effects of metabolically active substances secreted by fat on muscle strength, and the body size advantage of moderate amounts of abdominal fat may also enhance grip strength [37]. However, higher leptin concentrations are associated with an increased risk of frailty in older adults [38]. In a longitudinal study of middle-aged women, higher leptin concentrations predicted poorer mobility [39]. In addition, it has been shown that leptin levels are higher in patients with sarcopenic visceral obesity than in patients with sarcopenia alone [40]. Therefore, when BRI increased to the inflection point, the negative effect of fat on muscle strength intensified with further increases in fat and leptin concentrations, causing the increase in HGS to plateau. Our findings demonstrate the clinical utility of BRI as a screening tool for muscle health in public health practice. The identified threshold of 3.42 distinguishes two population subgroups requiring differentiated interventions: individuals with BRI < 3.42 (particularly aging adults and clinically vulnerable groups) may benefit from community programs integrating nutritional support and resistance training to reduce sarcopenia risk, while those with BRI ≥ 3.42 should receive combined aerobic-strength exercise and high-protein diets to concurrently address obesity and muscle preservation. Public health systems could improve sarcopenic obesity detection by incorporating BRI into routine assessments alongside traditional measures like BMI. Future research must validate this threshold across diverse populations and evaluate its clinical workflow integration.

BRI was negatively correlated with MQI, suggesting that the negative impact of obesity on muscle mass may receive multiple potential mechanisms. In obese individuals, hypertrophic adipocytes secrete interleukins (IL-6 and IL-1β), which inhibit the expression of contractile proteins in myotubes, leading to decreased muscle mass. Visceral adipocytes are more likely to induce this effect compared to subcutaneous fat [41]. A review indicated that the proportion of type I fibers was negatively correlated with BMI, while the proportion of type IIX fibers was positively correlated, and the proportion of type IIX fibers was significantly higher in obese individuals than in lean body types, while the proportion of type I fibers was relatively low [42]. Another study showed that obesity interferes with calcium signaling and 5’-adenosine monophosphate-activated protein kinase (AMPK) activity, leading to a shift in muscle fibers from type 1 to type 2 [43]. Although obesity increases the need for muscles to support body weight, thereby boosting absolute strength and power, muscle mass is decreased, which is consistent with the results of the present study. Furthermore, the negative impact of chronic inflammation and fat accumulation on muscle mass cannot be ignored. Clinical evidence suggests that adipose tissue in obese individuals produces chronic low-grade inflammation, which affects the function of other organs and tissues and increases the risk of metabolic disease [44]. Higher levels of inflammatory markers further reduce muscle mass [45]. A cross-sectional study on dietary inflammatory index and muscle mass showed that higher dietary inflammatory index was significantly associated with lower muscle mass and muscle strength and was more pronounced in older adults [46]. Another cross-sectional study in US adults also found that elevated levels of the Systemic Immune Inflammatory Index (SII) were associated with an increased risk of low muscle mass [47]. Rapid accumulation of intramuscular fat may affect muscle mass through metabolic pathways [48]. In a study of stroke patients [49], muscle strength in ambulatory chronic stroke survivors was shown to be strongly associated with muscle mass and intramuscular fat content of the quadriceps. Increasing muscle mass and decreasing intramuscular fat in the quadriceps bilaterally contributed to muscle strength. Similarly, in patients with rheumatoid arthritis, low muscle density is associated with low muscle mass, excessive obesity, decreased strength and increased disability [50]. Thus increased abdominal fat due to elevated BRI is closely associated with decreased MQI and may be caused by a combination of muscle fiber type shifts, chronic inflammation, and fat accumulation. These factors not only influence MQI, but may also explain the suppression of the rate of increase in HGS by obesity after the BRI inflection point.

In subgroup analyses, there was a significant interaction effect of age on the relationship between BRI and HGS. for each unit increase in BRI, the increase in HGS was significantly higher in those under 40 years of age than in those over 40 years of age. The likely reason for this is that muscle in the under 40s is in a rapid developmental phase, when the anabolic effects of fat outweigh the catabolic effects that may lead to muscle loss later in life [27]. In this age group, muscles are more responsive to mechanical stress stimuli, and the increase in BRI not only enhances the mechanical load but also provides adequate nutritional support, and a combination of factors contributes to the significant growth of HGS. In people over 40 years of age, with aging, immune cells in skeletal and cardiac muscle release more pro-inflammatory mediators, leading to disruption of endothelial structure, impaired cellular processes such as mitochondrial activity, mitochondrial autophagy, and a gradual weakening of myocyte contractile properties [51]. Thus, although obesity still provides mechanical stress stimulation to muscles in older people, HGS growth is more limited compared to younger people. In a stratified analysis of diabetic patients, it was found that when BRI increased by one unit, the reduction in MQI was smaller in diabetic patients than in the nondiabetic population. This may be related to fat distribution characteristics. BRI is calculated based on waist circumference and height, and diabetic patients usually show more significant central obesity [52]. Therefore, for the same increase in BRI, diabetic patients may have a lower overall fat gain than the non-diabetic population, resulting in a smaller decrease in their MQI. In addition, a review on obesity and diabetes noted that overweight or obese individuals who are able to increase subcutaneous fat stores, especially in the hip and thigh regions, may have lower health risks than expected in the presence of excess energy intake [53]. BRI showed significant interactions with HGS and MQI with respect to education level and ethnicity. This may be due to the fact that highly educated individuals usually possess greater health awareness and pay more attention to nutrition, exercise, and health management [54]. In addition, the effect of BRI on HGS and MQI varied by race due to differences in physiological and genetic characteristics and socio-cultural background. Two other studies have also found significant differences in the effects of BRI on muscle health among older adults of different races and countries [55,56].

This study has the following strengths. First, the sample data were obtained from NHANES, a database with a large sample size and reliable data that accurately represent the characteristics of the entire national population. Second, we adjusted for confounders and performed subgroup analyses to ensure significant correlations between BRI and HGS and MQI. Finally, this is the first study to address the relationship of BRI with HGS and MQI in a U.S. population. Nonetheless, this study has limitations to acknowledge. First, the cross-sectional design precludes causal inference between BRI and HGS/MQI relationships, necessitating future longitudinal verification. Second, while we adjusted for key demographic and metabolic confounders using NHANES-recommended socioeconomic indices, residual confounding from unmeasured factors persists. Third, physical activity data may be subject to recall bias despite NHANES standardized protocols. Most critically, two database-related constraints require emphasis: (1) restricted generalizability due to excluding institutionalized populations and age truncation (20–59 years), potentially underrepresenting marginalized subgroups and older adults; (2) absence of systematic strength training data – a critical musculoskeletal health determinant that may confound observed associations. Finally, findings are contextually bound to U.S. residents; cross-cultural validation through multinational cohorts remains imperative.

## Conclusion

This study found that BRI and HGS showed an L-shaped curve, while it was negatively correlated with MQI. This finding has important implications for understanding the relationship between abdominal obesity and HGS and MQI, suggesting that BRI can be a practical tool for assessing HGS and MQI in clinical settings. Furthermore, maintaining a moderate BRI had a positive impact on HGS (inflection point of 3.42).

## Supporting information

S1 (XLS)

## Abbreviations:

BRI: Body round index
HGS: Handgrip strength
MQI: Muscle quality index
BMI: Body mass index
ASM: Attached skeletal muscle
PIR: Poverty income ratio
ANOVA: Analysis of variance
VEGFA: Vascular endothelial growth factor A
AMPK: Activated protein kinase
SII: Immune inflammatory index.
